# Dietary Intake of Sulforaphane-Rich Broccoli Sprout Extracts during Juvenile and Adolescence Can Prevent Phencyclidine-Induced Cognitive Deficits at Adulthood

**DOI:** 10.1371/journal.pone.0127244

**Published:** 2015-06-24

**Authors:** Yumi Shirai, Yuko Fujita, Ryota Hashimoto, Kazutaka Ohi, Hidenaga Yamamori, Yuka Yasuda, Tamaki Ishima, Hiroyuki Suganuma, Yusuke Ushida, Masatoshi Takeda, Kenji Hashimoto

**Affiliations:** 1 Division of Clinical Neuroscience, Chiba University Center for Forensic Mental Health, Chiba, Japan; 2 Molecular Research Center for Children’s Mental Development, United Graduate School of Child Development, Osaka University, Osaka, Japan; 3 Department of Psychiatry, Osaka University Graduate School of Medicine, Osaka, Japan; 4 Research & Development Division, Kagome Co. Ltd., Tochigi, Japan; Tokyo Metropolitan Institute of Medical Science, JAPAN

## Abstract

Oxidative stress and inflammation play a role in cognitive impairment, which is a core symptom of schizophrenia. Furthermore, a hallmark of the pathophysiology of this disease is the dysfunction of cortical inhibitory γ-aminobutyric acid (GABA) neurons expressing parvalbumin (PV), which is also involved in cognitive impairment. Sulforaphane (SFN), an isothiocyanate derived from broccoli, is a potent activator of the transcription factor Nrf2, which plays a central role in the inducible expressions of many cytoprotective genes in response to oxidative stress. Keap1 is a cytoplasmic protein that is essential for the regulation of Nrf2 activity. Here, we found that pretreatment with SFN attenuated cognitive deficits, the increase in 8-oxo-dG-positive cells, and the decrease in PV-positive cells in the medial prefrontal cortex and hippocampus after repeated administration of phencyclidine (PCP). Furthermore, PCP-induced cognitive deficits were improved by the subsequent subchronic administration of SFN. Interestingly, the dietary intake of glucoraphanin (a glucosinolate precursor of SFN) during the juvenile and adolescence prevented the onset of PCP-induced cognitive deficits as well as the increase in 8-oxo-dG-positive cells and the decrease in PV-positive cells in the brain at adulthood. Moreover, the *NRF2* gene and the *KEAP1* gene had an epistatic effect on cognitive impairment (e.g., working memory and processing speed) in patients with schizophrenia. These findings suggest that SFN may have prophylactic and therapeutic effects on cognitive impairment in schizophrenia. Therefore, the dietary intake of SFN-rich broccoli sprouts during the juvenile and adolescence may prevent the onset of psychosis at adulthood.

## Introduction

Cognitive impairment is observed in patients with a number of psychiatric diseases, including schizophrenia, major depressive disorder, bipolar disorder, generalized anxiety disorder, panic disorder, post-traumatic stress disorder, obsessive compulsive disorder, attention deficit hyperactivity disorder, and autism spectrum disorder [1]. Cognitive impairment is also a core feature of schizophrenia, often persisting even when psychotic symptoms have been treated successfully [2, 3]. Interestingly, studies on adolescents and young adults at a high risk of developing psychosis have demonstrated cognitive impairment before the onset of psychotic symptoms [4–6]. Since cognitive impairment is a prodromal symptom, early intervention may prevent the onset of psychosis at adulthood [7, 8].

Oxidative stress and inflammation play a key role in the pathophysiology of schizophrenia as well as cognitive impairment in patients with psychiatric diseases such as schizophrenia [7–15]. The potent antioxidant sulforaphane (SFN: 1-isothiocyanato-4-methylsulfinylbutane) is an organosulfur compound derived from a glucosinolate precursor found in cruciferous vegetables, such as broccoli [16–18]. The protection afforded by SFN is thought to be mediated via the activation of the NF-E2-related factor-2 (Nrf2) pathway and the subsequent up-regulation of phase II detoxification enzymes and antioxidant proteins through an enhancer sequence referred to as the electrophilic-responsive element or the antioxidant-responsive element (ARE)[18–20]. Under normal conditions, Nrf2 is repressed by Keap1 (Kelch-like erythroid cell-derived protein with CNC homology [ECH]-associated protein 1), which is an adaptor protein for the degradation of Nrf2 [21]. During oxidative stress, Nrf2 is derepressed and activates the transcription of cytoprotective genes [21]. Recently, we reported that SFN could prevent behavioral abnormalities and dopaminergic neurotoxicity in mice after the administration of the psychostimulant methamphetamine [22]. Subsequently, we also reported that SFN could attenuate behavioral abnormalities in mice after the administration of the *N*-methyl-D-aspartate (NMDA) receptor antagonist phencyclidine (PCP)[23], since a PCP model of schizophrenia has been accepted throughout the world. These findings suggest that SFN could be a potential therapeutic natural compound for neuropsychiatric diseases, including substance abuse and schizophrenia [7, 22, 23].

Considering the potent antioxidant and anti-inflammatory actions of SFN, we hypothesized that SFN might be useful for the prevention or treatment of cognitive impairment in patients with psychiatric diseases. First, we examined whether SFN had prophylactic and therapeutic effects on cognitive deficits in mice after the repeated administration of PCP. Second, we examined whether the dietary intake of 0.1% glucoraphanin (GF: a glucosinolate precursor of SFN) during the juvenile and adolescence could prevent the onset of PCP-induced cognitive deficits at adulthood. Finally, we examined the association between the *KEAP1* and *NRF2* genes and cognitive function in patients with schizophrenia.

## Materials and Methods

### Animals

Male ICR mice (4 or 6 weeks old) weighing 25–30 g were purchased from SLC Japan (Hamamatsu, Shizuoka, Japan). The mice were housed in clear polycarbonate cages (22.5×33.8×14.0 cm) in groups of 5 or 6 individuals under a controlled 12-h light/12-h dark cycle (lights on from 7:00 AM to 7:00 PM), with the room temperature kept at 23°C ± 1°C and the humidity at 55% ± 5% to acclimatize the mice before the behavioral experiments. The mice were given free access to water and food pellets (CE-2; CLEA Japan, Inc., Tokyo, Japan). The experimental procedure was approved by the Chiba University Institutional Animal Care and Use Committee (Permission number: 26–24).

### Prophylactic effect of SFN on PCP-induced cognitive deficits

The treatment protocol for repeated PCP administration to induce cognitive deficits in mice has been previously reported [24–26]. Forty-six mice (6 weeks old) were divided into the following four groups: a vehicle (10 mL/kg/day, i.p., water in 10% corn oil) + saline (10 mL/kg/day, s.c.) group; a SFN (30 mg/kg/day, i.p.; LKT Laboratories, Inc., St. Paul, MN) + saline (10 mL/kg/day, s.c.) group; a vehicle (10 mL/kg/day, i.p.) + PCP (10 mg/kg/day as a hydrochloride salt, s.c.; synthesized by K. Hashimoto) group; and a SFN (30 mg/kg/day, i.p.) + PCP (10 mg/kg/day, s.c.) group. The interval between the first injection and the second injection was 30 min. In this study, we used a 30 mg/kg dose of SFN in the mice, as this was the most effective dose in previously reported experiments evaluating PCP-induced hyperlocomotion and PPI deficits [23]. Treatment was performed for 10 days (once daily on days 1–5 and 8–12). The NORT was performed on days 15 and 16.

### Therapeutic effect of SFN on PCP-induced cognitive deficits

Thirty-eight mice (6 weeks old) were divided into the following four groups: a saline + vehicle group; a saline + SFN group; a PCP + vehicle group; and a PCP + SFN group. Saline (10 mL/kg/day, s.c.) or PCP (10 mg/kg/day, s.c.) was administered on days 1–5 and days 8–12. Subsequently, SFN (30 mg/kg/day, i.p.) or the vehicle (10 mL/kg/day, i.p., water in 10% corn oil) was administered once daily on days 15–28. The NORT was performed on days 29 and 30.

### Prophylactic effect of the dietary intake of 0.1% glucoraphanin (GF) during the juvenile and adolescence on PCP-induced cognitive deficits at adulthood

Food pellets (CE-2; Japan CLEA, Ltd., Tokyo, Japan) containing 0.1% glucoraphanin (GF) were prepared as follows. Broccoli sprout extract powder containing SFN precursor GF was industrially produced by KAGOME CO., LTD. In brief, broccoli sprout was grown from specially selected seeds (Brassica Protection Products LLC., Baltimore, MD) for 1 day after the germination. The 1 day broccoli sprout was plunged into boiling water and maintained at 95°C for 30 minutes, and the sprout residues was removed by filtration. The boiling water extract was mixed with a waxy corn starch dextrin and then spray dried to yield the broccoli sprout extract powder containing 135 mg (approx. 0.31 mmol) of GF per gram. For preparing the animal diet containing 0.1% GF (approx. 2.3 mmol GF per 1 kg-diet), the extract powder was mixed with a basal diet CE-2 (CLEA Japan Inc., Tokyo, Japan), and then pelletized at a processing facility (Oriental Yeast Co., ltd., Tokyo, Japan). The GF content in the diet was determined by high performance liquid chromatography as previously described [27, 28].

Forty-three mice (4 weeks old) were divided into a normal food pellet group and a 0.1% GF-containing pellet group. The mice were given free access to water and both food pellets specifically designed for mice for 4-weeks (days 1–28). Subsequently, the mice were divided into the four groups: (1) a normal food + vehicle (10 mL/kg/day, s.c.) group; (2) a normal food + PCP (10 mg/kg/day, s.c.) group; (3) a 0.1% GF-containing food + vehicle (10 mL/kg/day, s.c.) group; and (4) a 0.1% GF-containing food + PCP (10 mg/kg/day, s.c.) group. Saline (10 mL/kg/day, s.c.) or PCP (10 mg/kg/day, s.c.) was administered on days 29–33 and days 36–40, as reported previously (24). In addition, normal food (CE-2) was given to the four groups on days 29–44. The NORT was performed on days 43 and 44.

### Novel object recognition test (NORT)

The NORT was performed as previously reported [24–26]. The apparatus for this task consisted of a black open-field box (50.8 × 50.8 × 25.4 cm). Before the test, mice were habituated to the box for 3 days. During the training session, two objects (various objects were used that differed with respect to shape and color, but that were similar in size) were placed in the box at a 35.5 cm distance from each other, and in a symmetrical fashion, and each animal was allowed to explore the interior of the box for 10 min (5 min × 2). The Animals were considered to be investigating the object when the head of the animal was either facing the object and was located within an inch of the object, or if any part of the body, except for the tail, was touching the object. After the training, the mice ware immediately returned to their home cages, and the box and objects ware cleaned with 75% ethanol to avoid any possible pheromonal cues. The retention test session was carried out one day after the respective training sessions. During each retention test session, each mouse was placed back into the same box it had previously encountered, but in which one of the two objects used during training session had been replaced by a novel object. The mice ware then allowed to freely explore the interior for 5 min, and the time spent exploring each object was again recorded. Throughout the experiments, the objects were used in a counter-balanced manner in terms of their physical complexity. In order to measure memory performance, a preference index was used, i.e., the ratio of the amount of time the mouse spent exploring any one of the two objects (training session) or the novel object (retention session) to total time spent exploring both objects.

### Golgi staining

Golgi staining was performed using the FD Rapid GolgiStain Kit (FD Neuro Technologies, Inc., Columbia, MD, USA), following the manufacturer's instructions, as previously reported [29, 30]. Mice were deeply anesthetized with sodium pentobarbital, and brains were removed from the skull and rinsed in double distilled water. Brains were immersed in the impregnation solution, made by mixing equal volumes of Solution A and B, overnight and then stored in fresh solution, for 2 weeks in the dark. Brains were transferred into Solution C overnight and then stored in fresh solution at 4°C for 1 week, in the dark. Coronal brain sections (100 μm thickness) were cut on a cryostat (3050S, Leica Microsystems AG, Wetzlar, Germany), with the chamber temperature set at -20°C. Each section was mounted in Solution C, on saline-coated microscope slides. After absorption of excess solution, sections were dried naturally, at room temperature. Dried sections were processed following the manufacturer's instructions. Briefly, images of dendrites within medial prefrontal cortex (mPFC), hippocampal CA1, CA3, and dentate gyrus (DG), nucleus accumbens (NAc)-core, NAc-shell, striatum and ventral tegmental area (VTA) were captured using a 100× objective with a Keyence BZ-9000 GenerationⅡmicroscope (Osaka, Japan). Spine density in these regions was counted as previously reported [29–31]. For spine density measurements, all clearly evaluable areas containing 50–100 μm of secondary dendrites from each imaged neuron were used. To determine relative spine density, spines on multiple dendritic branches from a single neuron were counted to obtain an average spine number per 10 μm. For spine number measurements, only spines that emerged perpendicular to the dendritic shaft were counted. Two to Three neurons per section, three sections per animal and seven to eight animals were analyzed. The average value for each region, in each individual was obtained. These individual averages were then combined to yield a grand average for each region.

### Immunohistochemistry for 8-oxo-dG

Immunohistochemistry of 8-hydroxy-2'-deoxyguanosine (8-oxo-dG) was performed by the previous reports [32, 33] with a slight modification. Mice ware anesthetized with sodium pentobarbital (50 mg/kg) and perfused transcardially with 10 mL of isotonic saline, followed by 40 mL of ice-cold, 4% paraformaldehyde in 0.1%M phosphate buffer (pH 7.4). Brains ware removed from the skulls and postfixed overnight at 4°C in the same fixative. For the immunohistochemical analysis, 50 μm-thick serial, coronal sections of brain tissue were cut in ice-cold, 0.01M phosphate buffered saline (pH 7.5) using a vibrating blade microtome (VT1000s, Leica Microsystems AG, Wetzlar, Germany). The Vector^Ⓡ^ Mouse on Mouse (MOM) Immunodetection Kit (Catalog No. PK-2200, Vector Laboratories, Inc., Burlingame, CA) was used. Free-floating sections were treated with 0.3% H_2_O_2_ in 50 mM Tris-HCL saline (TBS) for 30 min and rinsed two times in TBS and were blocked in TBS containing 0.2% Triton X-100 (TBST), 0.1% bovine serum albumin (BSA), and the MOM Ig blocking reagent for 1 h at room temperature. The sections ware quickly washed two times in TBS. The samples were then incubated in MOM diluent (add 600 μL of Protein Concentrate stock solution to 7.5 mL of TBS) for 5 min at room temperature. The samples were then incubated with mouse anti-8-oxo-dG antibody (1:250; Catalog # 4354-MC-050, TREVIGEN, Gaithersburg, USA) in the MOM Diluent for 30 min. The sections were washed quickly two times in TBS and then processed using the avidin-biotin-peroxidase method. The sections were incubated in biotinylated anti-mouse IgG reagent in MOM diluent. The sections were washed two times in TBS and incubated with avidin-biotin-peroxidase complex in TBS (add 2 drops of Reagent A to 2.5 mL of TBS, mix, and add 2 drops of Reagent B, mix) for 10 min. The sections were rinsed two times. Sections ware incubated for 3 min in a solution of 0.25 mg/mL diaminobenzidine (DAB) containing 0.01% H_2_O_2_. Then, sections were mounted on gelatinized slides, dehydrated, cleared, and coverslipped under Permount^Ⓡ^ (Fisher Scientific, Fair Lawn, NJ, USA). The sections were imaged, and the staining intensity of 8-oxo-dG immunoreactivity in the middle prefrontal cortex (mPFC), hippocampus (CA1, CA3, DG) was analyzed using a light micro-scope equipped with a CCD camera (Olymups IX70) and the SCION IMAGE software package. Images of sections within the mPFC and hippocampal CA1, CA3, and DG were captured using a 100× objective with a Keyence BZ-9000 GenerationⅡmicroscope (Osaka, Japan).

### Immunohistochemistry for parvalbumin (PV)

Mice ware anesthetized with sodium pentobarbital (50 mg/kg) and perfused transcardially with 10 mL of isotonic saline, followed by 40 mL of ice-cold, 4% paraformaldehyde in 0.1%M phosphate buffer (pH 7.4). Brains ware removed from the skulls and postfixed overnight at 4°C in the same fixative. For the immunohistochemical analysis, 50 μm-thick serial, coronal sections of brain tissue were cut in ice-cold, 0.001M phosphate buffered saline (pH 7.5) using a vibrating blade microtome (VT1000s, Leica Microsystems AG, Wetzlar, Germany). Free-floating sections were treated with 0.3% H_2_O_2_ in 50 mM Tris-HCL saline (TBS) for 30min and ware blocked in TBS containing 0.2% Triton X-100 (TBST) and 1.5% normal serum for 1 h at room temperature. The samples ware then incubated for 24 h at 4°C with rabbit polyclonal anti-parvalbumin (PV) antibody (1:2,500, Swant, Bellinzona, Switzerland). The sections were washed three times in TBS and then processed using the avidin-biotin-peroxidase method (Vectastain Elite ABC, Vector Laboratories, Inc., Burlingame, CA, USA). Sections ware incubated for 3 min in a solution of 0.25 mg/mL DAB containing 0.01% H_2_O_2_. Then, sections were mounted on gelatinized slides, dehydrated, cleared, and cover slipped under Permount^Ⓡ^(Fisher Scientific, Fair Lawn, NJ, USA). The sections were imaged, and the staining intensity of PV immunoreactivity in the mPFC, hippocampus (CA1, CA3, DG) was analyzed using a light micro-scope equipped with a CCD camera (Olymups IX70) and the SCION IMAGE software package. Images of sections within mPFC and hippocampal CA1, CA3, DG regions were captured using a 100× objective with a Keyence BZ-9000 GenerationⅡmicroscope (Osaka, Japan).

### Gene analysis of the *NRF2* and *KEAP1* gene variants in humans

All subjects were biologically unrelated within the second-degree of relationship and of Japanese descent. Subjects were excluded if they had neurological or medical conditions that could potentially affect the central nervous system, as previously described [34, 35]. Cases were recruited from the Osaka University Hospital. Each patient with schizophrenia had been diagnosed by at least two trained psychiatrists according to the criteria of the *Diagnostic and Statistical Manual of Mental Disorders*, *Fourth Edition* (DSM-IV) based on the Structured Clinical Interview for DSM-IV (SCID). Controls were recruited through local advertisements at Osaka University. Psychiatrically, medically and neurologically healthy controls were evaluated using the non-patient version of the SCID to exclude individuals who had current or past contact with psychiatric services or received psychiatric medication. The mean age and gender ratio did not differ significantly between cases and controls (*P* > 0.10), while the years of education and estimated premorbid IQ were significantly lower in the patients with schizophrenia than the controls (*P* < 0.001) (Table 1). Written informed consent was obtained from all subjects after the procedures had been fully explained. This study was conducted in accordance with the World Medical Association’s Declaration of Helsinki and approved by the Research Ethical Committee of Osaka University (Permission number: 379).

**Table 1:** Demographic information for patients with schizophrenia and healthy controls.

	Schizophrenia	Control	
Variable	(n = 183)	(n = 385)	*P* value (z)
Age (years)	34.9 ± 12.3	37.0 ± 12.9	0.13 (-1.51)
Gender (male/female)	103/80	188/197	0.10 (2.76)^a^
Education (years)	13.9 ± 2.6	15.0 ± 2.2	**<0.001 (-4.45)**
Estimated premorbid IQ	101.1 ± 10.3	107.6 ± 8.0	**<0.001 (-7.10)**
CPZ-eq. (mg/day)	574.0 ± 549.7	-	-
Age at onset (years)	24.1 ± 9.3	-	-
Duration of illness (years)	10.8 ± 9.7	-	-
PANSS positive symptoms	19.7 ± 5.8	-	-
PANSS negative symptoms	20.1 ± 6.1	-	-
PANSS general psychopathology	43.6 ± 10.9	-	-

Data are the mean ± SD. Significant *P* values are shown in boldface.

^a^ χ^2^ test. Complete demographic information was not obtained for all subjects (estimated premorbid IQ in patients, n = 179; PANSS, n = 182). PANSS, Positive and Negative Syndrome Scale; CPZ-eq., chlorpromazine equivalent of total antipsychotics.

Venous blood was collected from the subjects, and genomic DNA was extracted from whole blood according to standard procedures. We selected three single nucleotide polymorphisms (SNPs); rs10930781 from *NRF2* gene and rs1048290 and rs11545829 from *KEAP1* gene. It has been reported that rs6721961 located in the promoter region of the *NRF2* gene affects the transcriptional activity of *NRF2* and the minor allele of rs6721961 SNP diminishes promoter activity of the gene [36]. As the rs6721961 did not exist in our genotyped Affymetrix Genome-Wide Human SNP Array 6.0 (Affymetrix, Santa Clara, CA)[37], we selected proxy SNP rs10930781 of the rs6721961 in Japanese population (JPT) (*r* ^*2*^ = 0.86). The rs10930781 was located in the intron region of the *NRF2* gene. The genotyping data was extracted from the array. Next, we selected two possible functional synonymous polymorphisms from exons in the *KEAP1* gene because no functional SNP affecting the *KEAP1* function has been reported. The selected rs1048290 and rs11545829 were Leu471Leu and Tyr537Tyr, respectively. As these SNPs of the *KEAP1* gene did not exist in the Affymetrix array or there was no proxy SNP around these SNPs in the array, these SNPs were genotyped using the TaqMan 5’-exonuclease allelic discrimination assay (Assay ID: rs1048290; C_9323035_1_, rs11545829; C_34043047_10, Applied Biosystems, Foster City, California, USA) as previously described [38,39]. Detailed information on the PCR conditions is available upon request. No deviation from the Hardy-Weinberg equilibrium (HWE) was detected in the examined SNPs in the patients or controls (*P*>0.05). According to these genotyping data, we divided subjects into two sets of the four groups that minimized minor allele carriers due to small sample size in some cells: i) major allele homozygotes (*NRF2* CC-*KEAP1* rs1048290 GG), *NRF2* major allele homozygotes plus *KEAP1* rs1048290 minor allele carriers (*NRF2* CC-*KEAP1* rs1048290 CG/CC), *NRF2* minor allele carriers plus *KEAP1* rs1048290 major allele homozygotes (*NRF2* CT/TT-*KEAP1* rs1048290 GG), and minor allele carriers (*NRF2* CT/TT-*KEAP1* rs1048290 CG/CC), ii) major allele homozygotes (*NRF2* CC-*KEAP1* rs11545829 CC), *NRF2* major allele homozygotes plus *KEAP1* rs11545829 minor allele carriers (*NRF2* CC-*KEAP1* rs11545829 CT/TT), *NRF2* minor allele carriers plus *KEAP1* rs11545829 major allele homozygotes (*NRF2* CT/TT-*KEAP1* rs11545829 CC), and minor allele carriers (*NRF2* CT/TT-*KEAP1* rs11545829 CT/TT).

To assess intellectual functions remarkably impaired in patients with schizophrenia [40], we used the full-scale IQ and the four subscales; Verbal Comprehension, Perceptual Organization, Working Memory and Processing Speed, of the Japanese version of the Wechsler Adult Intelligence Scale (WAIS)-third edition [41]. The subjects were assessed by trained clinical psychologists to obtain the scores on the WAIS.

### Statistical analysis

The animal experiment data was expressed as the mean ± standard error of the mean (S.E.M.). The statistical analysis was performed using PASW Statistics 20 (formerly SPSS statistics; SPSS, Tokyo, Japan). All data, including the behavioral study, Golgi staining, and immunohistochemistry results, were analyzed using two-way analyses of variance (ANOVA), followed by a *post hoc* Bonferroni/Dunn test. For all the analyses, *P* values of less than 0.05 were considered statistically significant.

The statistical analysis of the gene analysis was performed as follows. Differences in clinical characteristics between patients and controls were analyzed using *χ* ^*2*^ tests for categorical variables and the Mann-Whitney *U*-test for continuous variables using PASW Statistics 18.0 software (SPSS Japan Inc., Tokyo, Japan). Deviation from the HWE was tested separately in test cases and controls using *χ* ^*2*^ tests for goodness of fit using SNPAlyze V5.1.1 Pro software (DYNACOM; Yokohama, Japan). The effects of interaction between *NRF2* and *KEAP1* genetic variants (“rs10930781 and rs10482909” or “rs10930781 and rs11545829”) on intellectual abilities were analyzed by one-way analyses of covariance (ANCOVA). Genotype status of the *NRF2* and *KEAP1* variants was included in the analysis as an independent variable. Full-scale IQ or its subscales were included as a dependent variable. As intellectual abilities may be influenced by sex and years of education, these variables were corrected for as covariates. We did not include age as a covariate because IQ scores were already corrected for age. The significance level for all statistical tests was set at two-tailed *P* < 0.05.

## Results

### Prophylactic effect of SFN on cognitive deficits and dendritic spine density after the repeated administration of PCP

**Fig 1:**
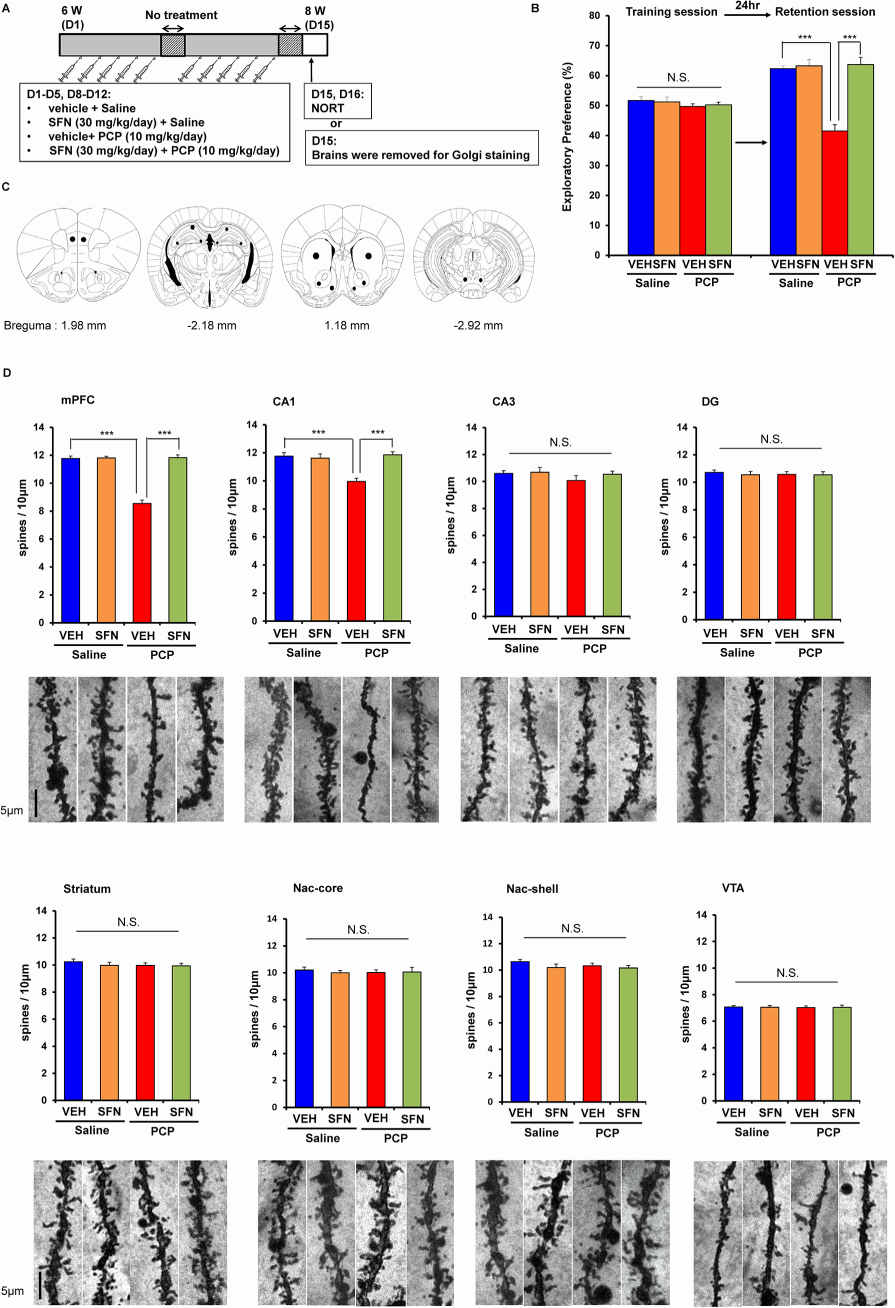
Prophylactic effect of SFN on PCP-induced cognitive deficits. (A): Schedule of PCP treatment, novel object recognition test (NORT), and Golgi staining. (B): On days 15 and 16, NORT was performed. Pretreatment with SFN significantly attenuated PCP-induced cognitive deficit in mice. The data show the mean ± S.E.M. (n = 11 or 12). (C): Brain regions of medial prefrontal cortex (mPFC), CA1, CA3, and dentate gyrus (DG) of hippocampus, striatum, Nucleus accumbens (NAc) shell and core, ventral tegmental area (VTA) were shown. (D): On day 15, brains of all groups were collected, and Golgi staining of all brain samples was performed. Repeated PCP administration significantly decreased the density of spine in the mPFC and CA1, but not CA3, DG, striatum, NAc shell and core, and VTA. Pretreatment with SFN significantly attenuated PCP-induced reduction of spine density in the mPFC and CA1. The data show the mean ± SEM (n = 7 or 8). ***P < 0.001, N.S. not significant.

Using the novel object recognition test (NORT), we previously reported that the repeated administration of PCP (10 mg/kg/day for 10 days) caused long-term cognitive deficits in mice (lasting more than 6 weeks after the final administration of PCP)[24]. First, we examined the prophylactic effect of SFN (30 mg/kg/day, administered 30 min before PCP administration) on PCP-induced cognitive deficits in mice (Fig 1A). In the training session, a two-way ANOVA analysis revealed no significant interaction (F [1,42] = 2.042, *P* = 0.160) (Fig 1B). However, in the retention session, a two-way ANOVA analysis revealed a significant interaction (F [1,42] = 11.05, *P* = 0.002) (Fig 1B). A *post hoc* Bonferroni test indicated that pretreatment with SFN significantly (*P* < 0.001) attenuated PCP-induced cognitive deficits in mice. In contrast, pretreatment with SFN did not alter cognition in the control (saline-treated) mice (Fig 1B).

Previous reports have demonstrated that the repeated administration of PCP causes a loss of dendritic spine density in the prefrontal cortex (PFC) of rats and monkeys [42–44]. In this study, we examined whether SFN affected alterations in the dendritic spines of the medial prefrontal cortex (mPFC), CA1, CA3, dentate gyrus (DG) of the hippocampus, striatum, nucleus accumbens (NAc), or ventral tegmental area (VTA) after the repeated administration of PCP. A two-way ANOVA analysis revealed interactions (mPFC: F [1,25] = 68.96, *P* < 0.001; CA1: F [1,25] = 9.891, *P* < 0.004; CA3, F [1,25] = 0.094, *P* = 0.762; DG: F [1,25] = 0.118, *P* = 0.734; striatum: F [1,25] = 0.344, *P* = 0.563; NAc-shell: F [1,25] = 0.655, *P* = 0.426; NAc-core: F [1,25] = 0.063, *P* = 0.804; VTA: F [1,25] = 0.040, *P* = 0.842). A *post hoc* Bonferroni test showed that the repeated administration of PCP (10 mg/kg/day for 10 days) significantly decreased the dendritic spine density in the mPFC (*P* < 0.001) and CA1 (*P* < 0.001), but not in the other regions. Furthermore, pretreatment with SFN significantly (*P* < 0.001) protected against the PCP-induced reduction in the spine density in these two regions (Fig 1C and Fig 1D).

### Prophylactic effect of SFN on oxidative stress in the brain after the repeated administration of PCP

**Fig 2:**
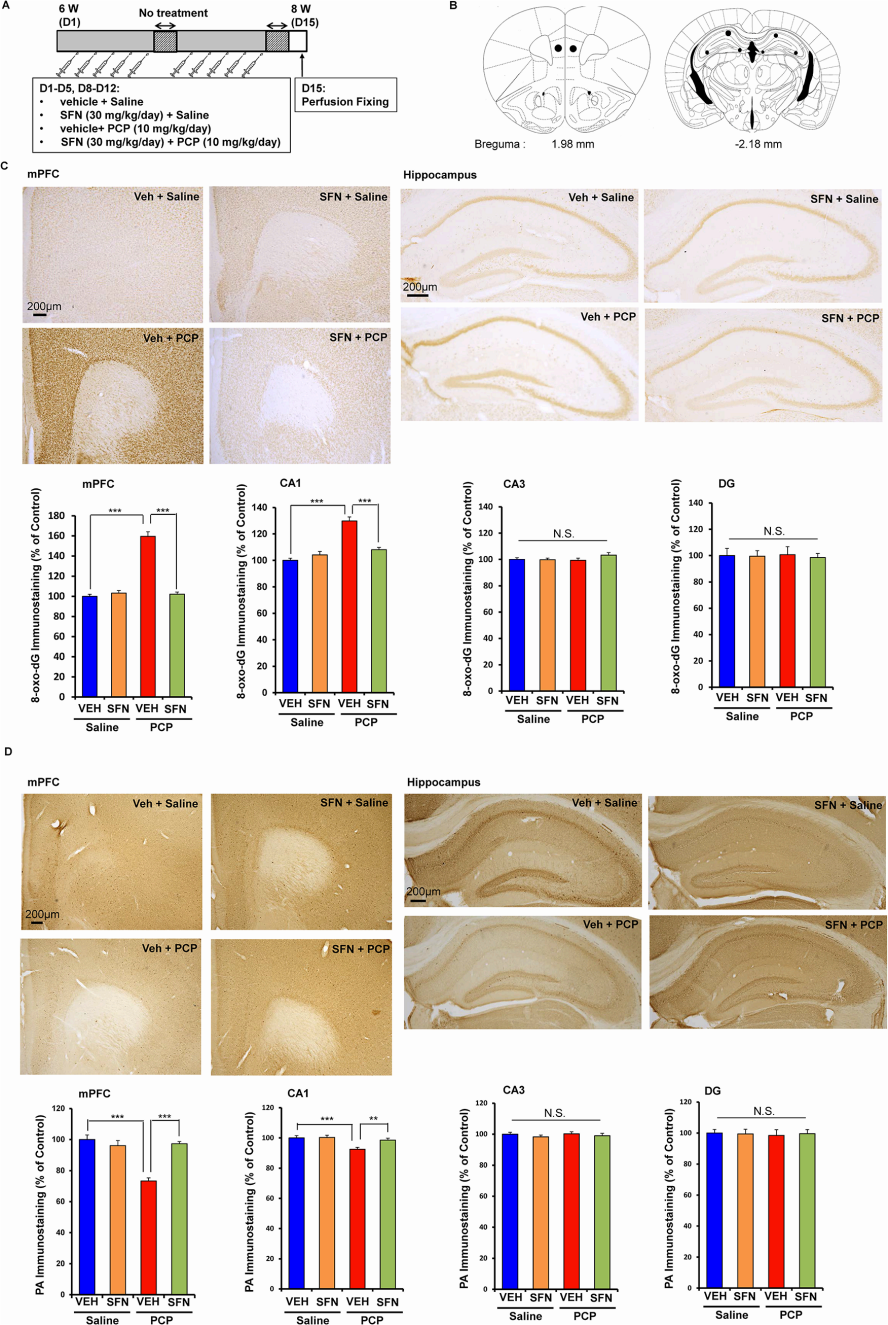
Prophylactic effect of SFN on PCP-induced alterations in 8-oxo-dG immunostaining and PV-positive immunostaining in the brain. (A): Treatment schedule and immunohistochemistry. On day 15, all mice were perfused. Then immunohistochemistry was performed. (B): Brain regions of medial prefrontal cortex (mPFC), CA1, CA3, and dentate gyrus (DG) of hippocampus, were shown. (C): Repeated PCP administration significantly increased 8-oxo-dG immunostaining in the mPFC and CA1, but not CA3 and DG. Pretreatment with SFN significantly attenuated PCP-induced increases of 8-oxo-dG immunostaining in the mPFC and CA1. The data show the mean ± SEM (n = 6 or 7). (C): Repeated PCP administration significantly decreased PV-positive immunostaining in the mPFC and CA1, but not CA3 and DG. Pretreatment with SFN significantly attenuated PCP-induced decreases of PV-positive immunostaining in the mPFC and CA1. The data show the mean ± SEM (n = 6 or 7). **P < 0.01, ***P < 0.001, N.S. not significant.

Oxidative stress plays a role in the pathophysiology of schizophrenia as well as cognitive impairment in patients with psychiatric diseases [7–11]. To examine the effect of SFN on oxidative stress, we performed immunohistochemistry for 8-hydroxy-2'-deoxyguanosine (8-oxo-dG) (a marker of DNA oxidative damage) in the mouse brain (Fig 2A). The repeated administration of PCP (10 mg/kg/day for 10 days) significantly increased the proportion of 8-oxo-dG-positive cells in regions of the mouse brain. A two-way ANOVA showed interactions for the examined regions (mPFC: F [1,21] = 104.2, *P* < 0.001; CA1: F [1,21] = 57.732, *P* < 0.001; CA3, F [1,21] = 0.625, *P* = 0.438; DG: F [1,21] = 0.625, *P* = 0.438). A *post hoc* Bonferroni test showed that the repeated administration of PCP (10 mg/kg/day for 10 days) significantly increased the proportion of 8-oxo-dG-positive cells in the mPFC (*P* < 0.001) and CA1 (*P* < 0.001), but not in the other regions. Pretreatment with SFN significantly protected against the PCP-induced increase in the 8-oxo-dG-positive cells in these two regions (Fig 2B and Fig 2C).

Alterations in parvalbumin (PV)-positive cells in the brain are known to play a role in cognitive impairment in schizophrenia [32,33,45]. A two-way ANOVA showed significant interactions for two regions (mPFC: F [1,21] = 24.956, *P* < 0.001; CA1: F [1,21] = 10.517, *P* = 0.004; CA3: F [1,21] = 0.119, *P* = 0.733; DG: F [1,21] = 0.028, *P* = 0.869). A *post hoc* Bonferroni test showed that the repeated administration of PCP (10 mg/kg/day for 10 days) significantly decreased the proportion of PV-positive cells in the mPFC (*P* < 0.001) and CA1 (*P* < 0.01), but not in the other regions. Furthermore, pretreatment with SFN significantly protected against the PCP-induced decrease in PV-positive cells in these two regions (Fig 2B and Fig 2D).

### PCP-induced cognitive deficits improved after the subsequent subchronic administration of SFN

**Fig 3:**
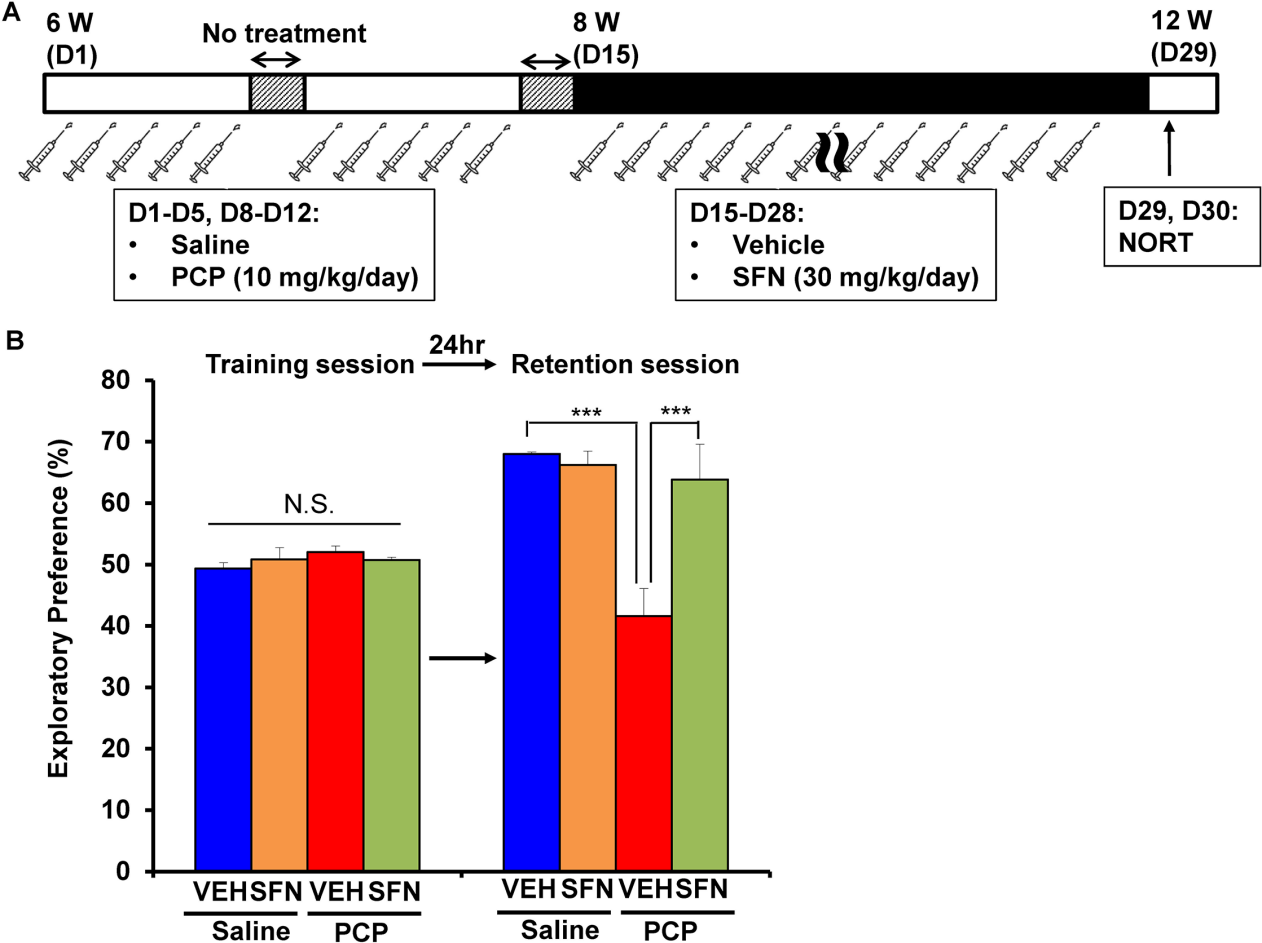
Therapeutic effect of SFN on PCP-induced cognitive deficits in mice. (A): Schedule of treatment and NORT. From days 1–5 and 8–12, saline (10 ml/kg/day) or PCP (10 mg/kg/day) was administered into mice. Subsequently, vehicle (10 ml/kg/day) or SFN (30 mg/kg/day) was administered from days 15–28. On day 29 and 30, NORT was performed. (B): PCP-induced cognitive deficit in mice were significantly improved by subsequent subchronic administration of SFN. The data show the mean ± S.E.M. (n = 8–11). ***P < 0.001, N.S. not significant.

To examine the therapeutic effect of SFN, we examined whether SFN can improve PCP-induced cognitive deficits in mice (Fig 3A). PCP (10 mg/kg/day for 10 days)-induced cognitive deficits in mice improved significantly after the subsequent subchronic (14 days) administration of SFN (30 mg/kg/day) (Fig 3B). In the training session, the exploratory preferences of mice in four groups were not different (interaction: F [1,34] = 3.185, *P* = 0.083) (Fig 3B). However, in the retention session, a two-way ANOVA analysis revealed that the exploratory preferences among the four groups differed significantly (interaction: F [1,34] = 14.771, *P* < 0.001) (Fig 3B). A *post hoc* Bonferroni test indicated that the exploratory preference of the PCP-treated group increased significantly (*P* < 0.001) after the subchronic (14 days) administration of SFN (30 mg/kg/day) (Fig 3B). In contrast, the subchronic (14 days) administration of SFN (30 mg/kg/day) did not alter cognition in control (saline-treated) mice (Fig 3B).

### Dietary intake of 0.1% GF-containing food during the juvenile and adolescence prevented PCP-induced cognitive deficits and oxidative stress at adulthood

**Fig 4:**
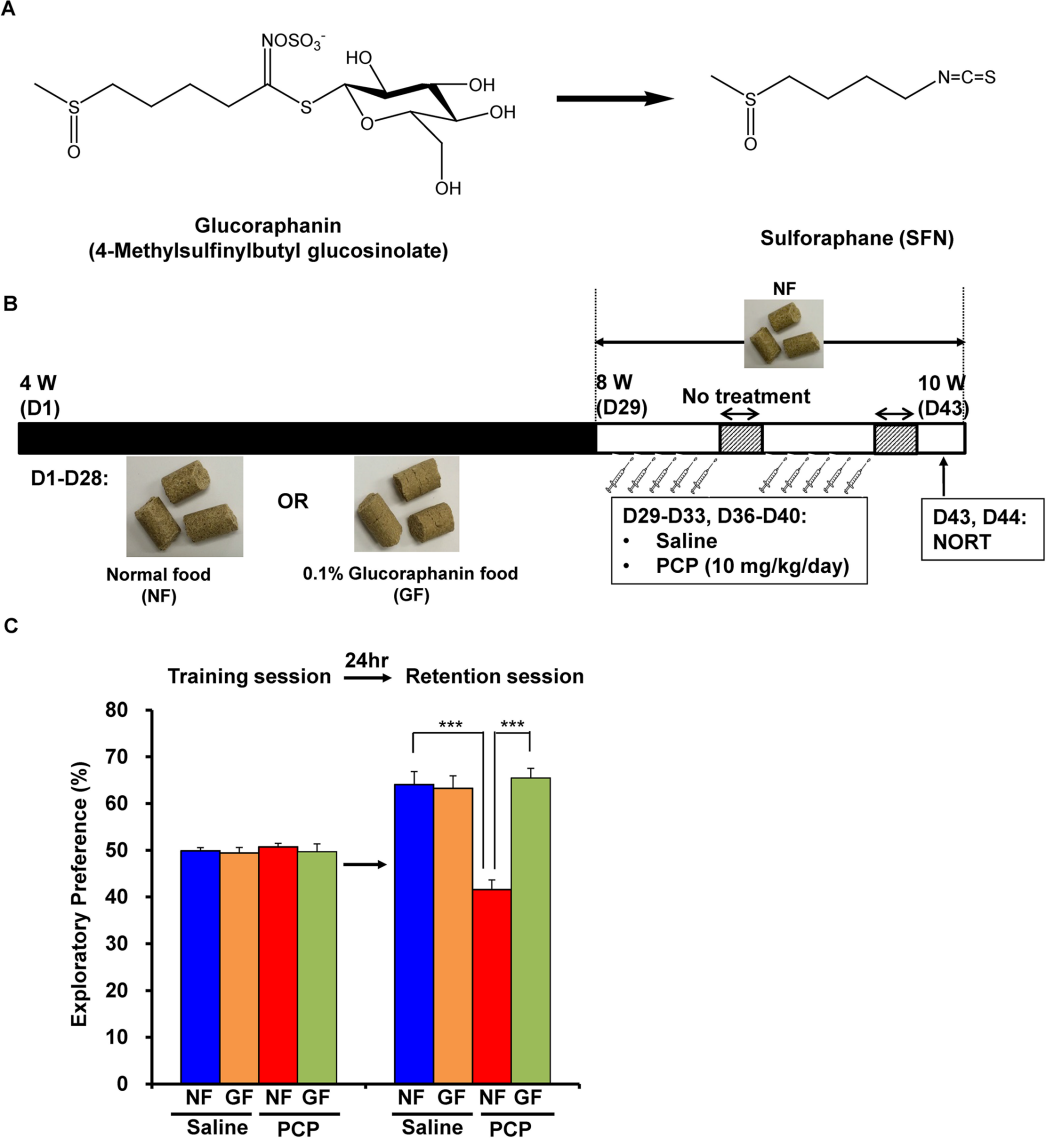
Prophylactic effect of dietary GF food during juvenile and adolescence on PCP-induced cognitive deficits in mice at adulthood. (A): Chemical structure of glucoraphanine (GF) and sulforaphane (SFN). Glucoraphanine (GF) is metabolized to SFN in the body. (B): Schedule of treatment and NORT. From days 1 (4-week olds)– 28 (8-week olds), normal food (NF) or 0.1% GF food (GF) was administered into mice. From days 29 (8-week olds), normal food was administered to all mice. Subsequently, saline (10 ml/kg/day) or PCP (10 mg/kg/day) was administered from days 29–33 and days 36–40. On day 43 and 44, NORT was performed. (C): Dietary 0.1% GF food during days 1–28 significantly attenuated PCP-induced cognitive deficit in mice at adulthood (10-week olds). The data show the mean ± S.E.M. (n = 10–12). ***P < 0.001, N.S. not significant.

Since cognitive impairment can be detected at a prodromal stage, we examined whether the dietary intake of SFN during the juvenile and adolescence might prevent the onset of PCP-induced cognitive deficits at adulthood. In this study, we used 0.1% GF-containing food pellets, since GF is the glucosinolate precursor of SFN (Fig 4A). We examined whether the dietary intake of 0.1% GF-containing food pellets between the ages of 4 and 8 weeks could prevent PCP (10 mg/kg/day for 10 days)-induced cognitive deficits at adulthood (10 weeks) (Fig 4B). In the training session, the exploratory preferences among four groups were not different (interaction: F [1,39] = 0.205, *P* = 0.654) (Fig 4B). However, in the retention session, a two-way ANOVA analysis revealed that the exploratory preferences among the four groups differed significantly (interaction: F [1,39] = 16.490, *P* < 0.001) (Fig 4B). A *post hoc* Bonferroni test indicated that the exploratory preference of the PCP-treated group increased significantly (*P* < 0.001) after the dietary intake of 0.1% GF-containing pellets (Fig 4C). In contrast, the dietary intake of 0.1% GF-containing pellets between 4 and 8 weeks did not alter cognition in control (saline-treated) mice (Fig 4C).

**Fig 5:**
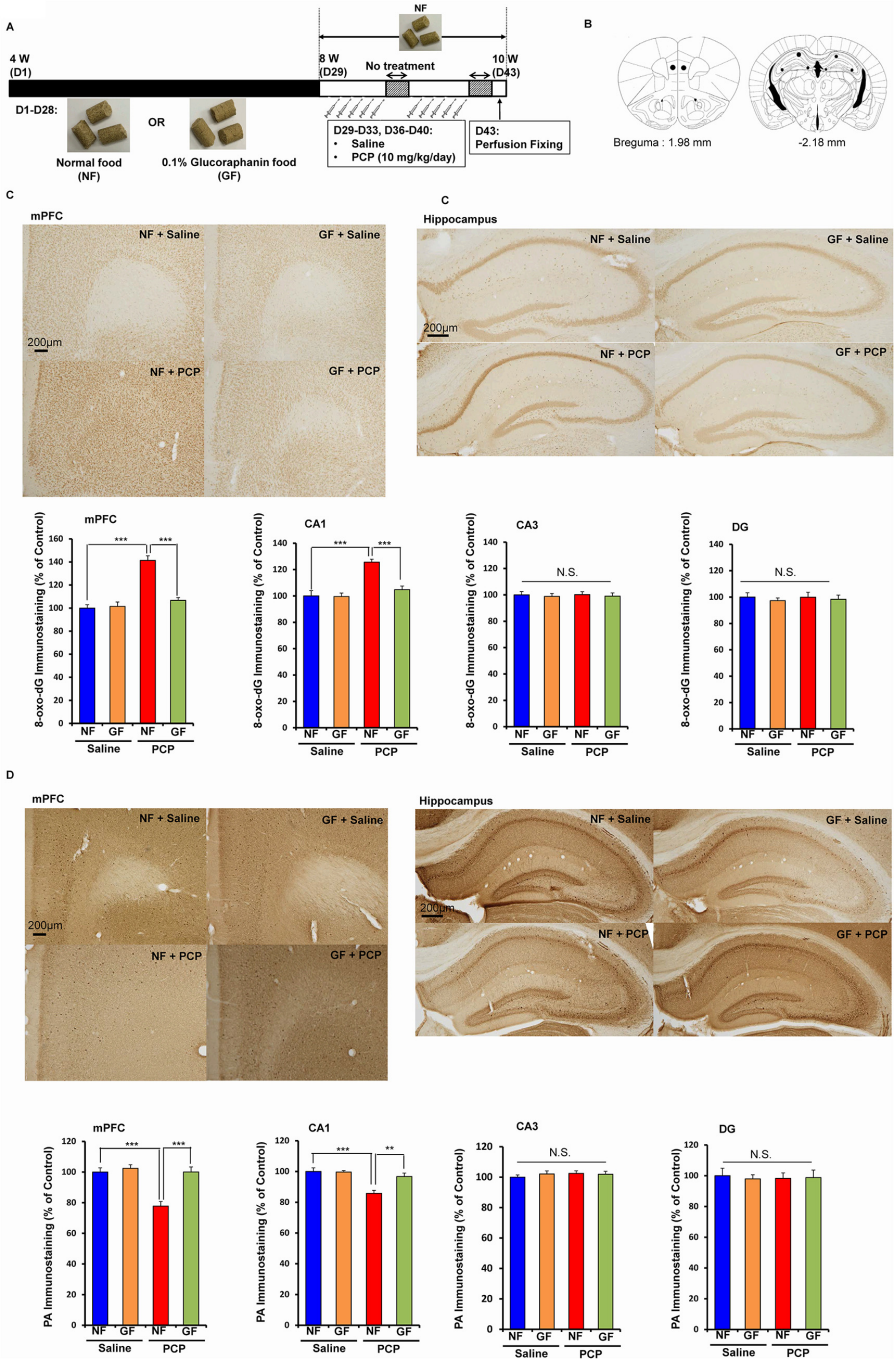
Prophylactic effect of dietary GF food during juvenile and adolescence on PCP-induced oxidative stress and reduction of PV-positive cells in the brain at adulthood. (A): Schedule of treatment, and immunohistochemistry. From days 1 (4-week olds)– 28 (8-week olds), normal food (NF) or 0.1% glucoraphanine food (GF) was administered into mice. From days 29 (8-week olds), normal food was administered to all mice. Subsequently, saline (10 ml/kg/day) or PCP (10 mg/kg/day) was administered from days 29–33 and days 36–40. On day 43, all mice were perfused. (B): Brain regions of mPFC, CA1, CA3, and dentate gyrus (DG) of hippocampus, were shown. (C): Repeated PCP administration significantly increased 8-oxo-dG immunostaining in the mPFC and CA1, but not CA3 and DG. Dietary intake of 0.1% GF from 4-week old to 8-week olds significantly attenuated PCP-induced increases of 8-oxo-dG immunostaining in the mPFC and CA1 at 10-week olds. The data show the mean ± SEM (n = 5–7). (D): Repeated PCP administration significantly decreased PV-positive immunostaining in the mPFC and CA1, but not CA3 and DG. Dietary intake of 0.1% GF from 4-week old to 8-week olds significantly attenuated PCP-induced decreases of PV-positive immunostaining in the mPFC and CA1 at 10-week olds. The data show the mean ± SEM (n = 5–7). **P < 0.01, ***P < 0.001, N.S. not significant.

To examine the effect of the dietary intake of 0.1% GF-containing pellets during the juvenile and adolescence on PCP-induced oxidative stress, we performed 8-oxo-dG immunohistochemistry in the mouse brain (Fig 5A and Fig 5B). A two-way ANOVA showed interactions for the examined regions (mPFC: F [1,20] = 26.299, *P* < 0.001; CA1: F [1,20] = 12.224, *P* = 0.002; CA3, F [1,20] = 0.901, *P* = 0.354; DG: F [1,20] = 0.482, *P* = 0.496). A *post hoc* Bonferroni test showed that the repeated administration of PCP (10 mg/kg/day for 10 days) significantly increased the proportion of 8-oxo-dG-positive cells in the mPFC (*P* < 0.001) and CA1 (*P* < 0.001), but not in the CA3 and DG. The dietary intake of 0.1% GF-containing pellets significantly protected against the PCP-induced increase in the 8-oxo-dG-positive cells in these two regions (Fig 5C).

To examine the effect of the dietary intake of 0.1% GF-containing pellets during the juvenile and adolescence on PCP-induced reduction of PV-positive cells, we performed PV-immunohistochemistry in the mouse brain. A two-way ANOVA showed significant interactions for two regions (mPFC: F [1,20] = 18.897, *P* < 0.001; CA1: F [1,20] = 7.156, *P* = 0.015; CA3: F [1,20] = 0.159, *P* = 0.694; DG: F [1,20] = 0.034, *P* = 0.856). A *post hoc* Bonferroni test showed that the repeated administration of PCP (10 mg/kg/day for 10 days) significantly decreased the proportion of PV-positive cells in the mPFC (*P* < 0.001) and CA1 (*P* < 0.001), but not in the CA3 and DG. Furthermore, the dietary intake of 0.1% GF-containing pellets significantly (mPFC: P < 0.001, CA1: P < 0.01) protected against the PCP-induced decrease in PV-positive cells in these two regions (Fig 5D).

### Effect of genetic interaction between NRF2 and KEAP1 variants on intellectual abilities

**Fig 6:**
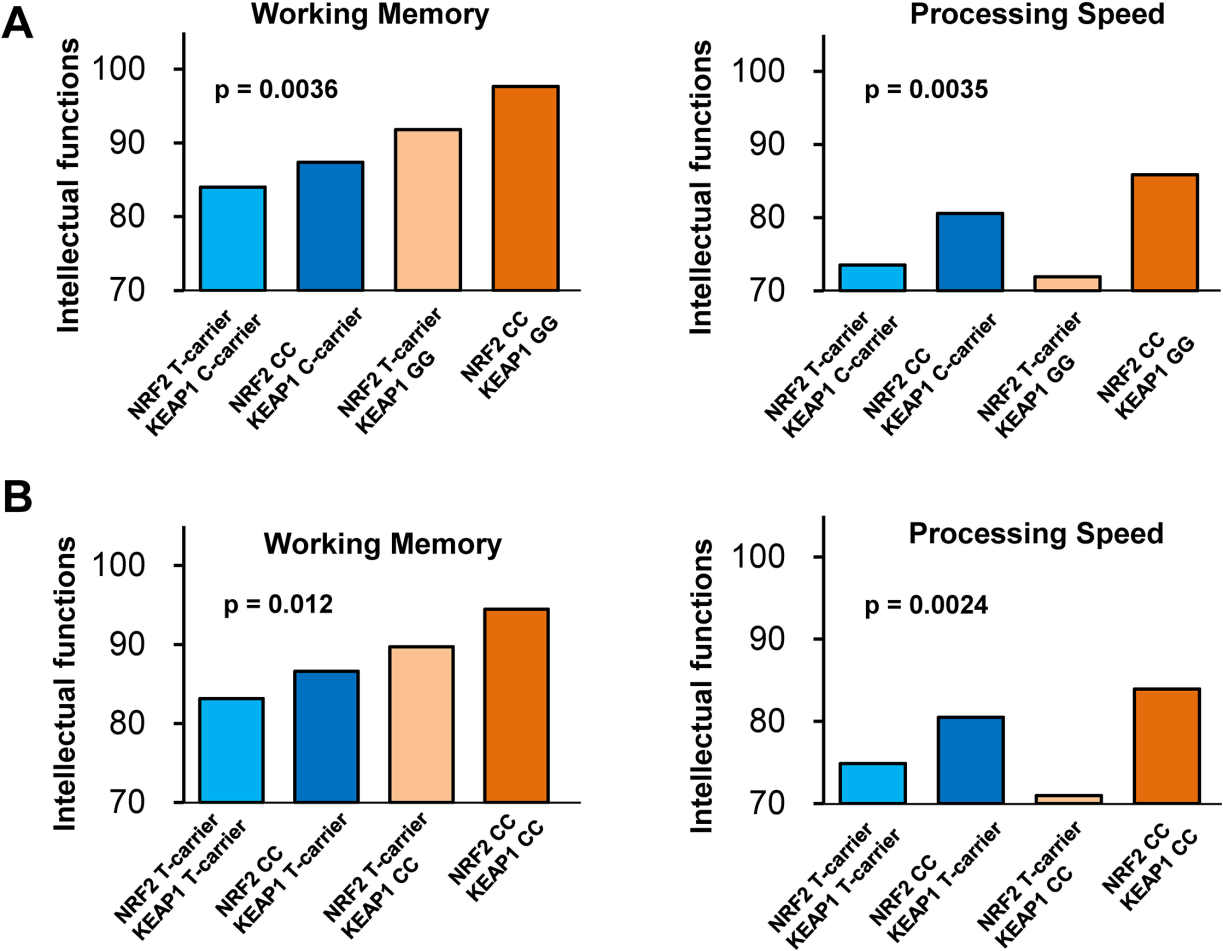
Interaction between *NRF2* gene variant and *KEAP1* gene variants on intellectual ability in patients with schizophrenia. (A): There was a significant (P = 0.0036) epistatic effect of *NRF2* gene (rs10930781) and *KEAP1* gene variant (rs1048290) on working memory in patients with schizophrenia, but not controls (S1 Table). On the other hand, a significant (*P* = 0.0035) effect of these variants on processing speed was due to the *NRF2* gene variant. (B): There was a significant (*P* = 0.012) epistatic effect of *NRF2* gene (rs10930781) and *KEAP1* gene variant (rs11545829) on working memory in schizophrenia, but not controls (S2 Table). On the other hand, a significant effect (*P* = 0.0024) of these variants on processing speed in schizophrenia was due to the *NRF2* gene variant (S2 Table).

Analysis of *NRF2* and *KEAP1* genes was conducted with 183 patients with schizophrenia and 385 healthy subjects (Table 1). Since evidence exists of a biological interaction between the NRF2 and KEAP1 proteins [21], the combined effect of *NRF2* and *KEAP1* genetic variants might influence the impairments in intellectual functions observed in schizophrenia. In this study, we selected possible functional variants: rs10930781 in the *NRF2* gene, and rs1048290 or rs11545829 in the *KEAP1* gene in patients with schizophrenia and healthy controls (S1 Table and S2 Table). We first investigated the genetic interactions between rs10930781 and rs1048290 on intellectual functions separately in patients with schizophrenia and healthy subjects (Table 1). In patients, we found that these variants had significant effects on working memory (F_1,177_ = 4.7, *P* = 0.0036) and processing speed (F_1,177_ = 4.7, *P* = 0.0035) (Fig 6A and S1 Table). A *post hoc* analysis on working memory found that patients with a CC–GG genotype at rs10930781 and rs1048290 scored higher than those with a T carrier–C carrier genotype (*P* = 0.00043) or those with a CC–C carrier genotype (*P* = 0.0075). On the other hand, a *post hoc* analysis on processing speed found that patients with a CC–GG genotype scored higher than those with a T carrier–GG genotype (*P* = 0.0040) or those with a T carrier–C carrier genotype (*P* = 0.0018), and patients with a CC–C carrier genotype scored marginally higher than those with a T carrier–GG genotype (*P* = 0.039) or those with a T carrier–C carrier genotype (*P* = 0.029). These findings indicated that the interaction of these variants on working memory exhibited an epistatic gene effect, while the interaction on processing speed was caused by the effect of the *NRF2* gene variant. No effect of these variants on full-scale IQ, verbal comprehension, or perceptual organization (*P* > 0.05) was observed. In healthy controls, no effect of these variants on any score was seen (*P* > 0.05) (S1 Table).

When we investigated the interaction of rs10930781 in the *NRF2* gene and the other single nucleotide polymorphism (rs11545829) in the *KEAP1* gene on intellectual functions in patients with schizophrenia, we found similar results to the above interactions observed for working memory (*P* = 0.012) and processing speed (*P* = 0.0024) (Fig 6B and S2 Table **)**. The individual effects of each variant on intellectual abilities are shown (S2 Table, S3 Table and S4 Table). These findings may support a biological interaction between the NRF2 and KEAP1 proteins.

## Discussion

In the present study, we demonstrated that SFN has prophylactic and therapeutic effects on PCP-induced cognitive deficits in mice. First, pretreatment with SFN was able to prevent the onset of PCP-induced cognitive deficits in mice, suggesting that dietary SFN may prevent cognitive impairment associated with environmental events (e.g., oxidative stress and inflammation) in humans. Second, PCP-induced cognitive deficits in mice were improved by the subsequent subchronic administration of SFN, suggesting that SFN might improve cognitive impairments in patients with schizophrenia. Previously, we reported that PCP-induced cognitive deficits in mice could be improved by the subsequent subchronic administration of the atypical antipsychotic drug clozapine, but not the typical antipsychotic drug haloperidol, indicating that PCP-induced cognitive deficits identified using NORT could represent a potential animal model for atypical antipsychotic activity [24]. Therefore, it should be noted that SFN exhibited an atypical antipsychotic activity in this model. Third, the dietary intake of 0.1% GF (a precursor of SFN) during the juvenile and adolescence was able to prevent subsequent PCP-induced cognitive deficits, oxidative stress and the reduction of PV-positive cells at adulthood, indicating that SFN intake during the juvenile and adolescence was able to protect against PCP-induced cognitive deficits and oxidative stress at adulthood. Fourth, a genetic analysis showed an epistatic interaction between *NRF2* and *KEAP1* gene variants on working memory in schizophrenia. Taken together, these findings suggest that the Keap1—Nrf2 system may play a role in the cognitive impairment that is observed in schizophrenia, and that SFN, an activator of Nrf2, may have preventive or therapeutic effects on cognitive impairment in patients with psychiatric diseases such as schizophrenia.

Long-term PCP abuse in humans is known to lead to enduring cognitive impairment [46]. In rodents, the repeated administration of PCP causes long-term cognitive deficits [24–26, 47]. In the present study, we found that SFN exhibited prophylactic and therapeutic effects in a PCP-induced cognitive deficits model. Very recently, we found that supplementation with SFN-rich broccoli sprout extract for 8 weeks was effective for the treatment of cognitive impairment in medicated patients with schizophrenia, although other scores (such as psychotic symptoms) were not altered [48]. Furthermore, a recent randomized, double-blinded, placebo-controlled study demonstrated that treatment with SFN-rich broccoli sprout extract significantly improved social interaction, abnormal behavior, and verbal communication in young men with autism spectrum disorder [49]. Glucoraphanin (GF), a glucosinolate precursor of SFN, is widely consumed in cruciferous plant-rich diets; therefore, SFN is considered to have a low toxicity, and its administration in humans is well tolerated [18, 49–51]. It is reported that SFN readily crosses the blood-brain barrier of mouse after i.p. administration [52], suggesting that SFN in the brain can improve PCP-induced cognitive deficits. Together, these results suggest that SFN-rich broccoli sprout extract could have a potential therapeutic effect in patients with a number of psychiatric diseases including schizophrenia and autism spectrum disorder, since patients with these psychiatric diseases exhibit cognitive impairment [1].

Changes in dendritic length and spine density in the PFC and hippocampus are thought to contribute to the neurobiology of cognitive impairment in patients with schizophrenia [53–56]. In this study, we observed a loss of spine density in the mPFC and CA1 of hippocampus in PCP-treated mice, consistent with the findings of a previous report [42]. As the loss of dendritic spine density in the PFC and the hippocampus may contribute, at least in part, to PCP-induced cognitive deficits [47], the protective effects of SFN on the PCP-induced loss of spine density in these two regions may contribute to its beneficial effect on cognition.

Neurodevelopment during early adolescence is a key stage during maturation, with various structural, neurochemical, and molecular changes taking place in response to genetic and environmental cues. The formation of new neuronal connections during early adolescence also means a high level of vulnerability to pathologic insults ranging from stress to dietary deficiencies [57, 58]. The nutritional status during early adolescence has a great impact on the onset and severity of psychiatric diseases at adulthood [58]. In the past decade, increasing interest in the potential benefits of early intervention for psychiatric diseases, such as schizophrenia, has been seen. Subjects at a high risk of developing psychosis exhibit cognitive impairments, compared with healthy subjects [6]. Approximately one-third of subjects at a high risk develop psychosis within three years, and most are diagnosed as having schizophrenia [59]. In this study, we found that the dietary intake of SFN-rich food during the juvenile and adolescence was capable of preventing PCP-induced cognitive deficits and oxidative stress at adulthood in mice. Although the precise mechanism underlying the preventive effect of SFN-rich food is currently unclear, the dietary intake of SFN-rich foods may be capable of regulating gene expression through epigenetic mechanisms. Since SFN has potent antioxidant and anti-inflammatory effects, SFN may prevent the onset of psychosis in subjects at a high risk and who exhibit oxidative stress and inflammation. Therefore, a randomized, double-blinded, placebo-controlled study of the dietary intake of SFN-rich foods in subjects at a high risk of psychosis is of great interest.

The identification of gene-gene interactions is a hot topic in genome-wide association studies of psychiatric diseases, and epistatic gene-gene interactions may contribute to complex human traits as well as the heritability of complex multi-genetic disorders, such as psychiatric diseases [60, 61]. For example, Tan et al. [62] reported an apparent epistatic interaction between the catechol-*O*-methyltransferase (*COMT*) gene and the metabotropic glutamate receptor mGluR3 (*GRM3*) gene on the engagement of the prefrontal cortex during working memory, indicating an epistatic effect of these two genes in human cortical circuits that have been implicated in the working memory dysfunction that is observed in schizophrenia. In the present study, we observed an epistatic effect of the *NRF2* gene and the *KEAP1* gene on the impairment of working memory and processing speed in patients with schizophrenia. Given the crucial role of the Keap1-Nrf2 complex in both oxidative stress and inflammation [21, 63], the epistatic effect of these two genes on cognitive impairment in schizophrenia is of great interest.

In conclusion, the present study suggested that SFN had prophylactic and therapeutic effects in an animal model of cognitive deficits and that the dietary intake of GF during the juvenile and adolescence was capable of preventing the onset of cognitive deficits at adulthood. Furthermore, we observed an epistatic gene effect of the *NRF2* and *KEAP1* genes on cognitive impairment in patients with schizophrenia. Taken together, these results suggest that SFN-rich broccoli sprout extract could potentially be used for the treatment of cognitive impairment, since it is a naturally occurring compound found in cruciferous vegetables. Finally, further study of the dietary intake of SFN (or SFN-rich broccoli sprout extract) in subjects at a high risk of developing psychosis is needed to study the possible prophylactic effects of SFN.

## Supporting Information

S1 Table Genetic interaction between rs10930781 and rs1048290 on intellectual functions in patients with schizophrenia and healthy subjects. (PDF)

S2 Table Genetic interaction between rs10930781 and rs11545829 on intellectual functions in patients with schizophrenia and healthy subjects. (PDF)

S3 Table Effect of rs10930781 genotype on intellectual ability. (PDF)

S4 Table Effect of rs1048290 genotype on intellectual ability. (PDF)

S5 Table Effect of rs1048290 genotype on intellectual ability. (PDF)
