# Supplementary material for: Dietary Intake of Sulforaphane-Rich Broccoli Sprout Extracts during Juvenile and Adolescence Can Prevent Phencyclidine-Induced Cognitive Deficits at Adulthood
Source: PLoS One. 2015 Jun 24;10(6):e0127244. doi: 10.1371/journal.pone.0127244 (PMC4479552; doi:10.1371/journal.pone.0127244)
Supplement: S1 Table — (PDF) [file pone.0127244.s001.pdf]

Table S1. Genetic interaction between rs10930781 and rs1048290 on intellectual functions in patients with schizophrenia and healthy subjects

Variables	NRF2 T carrier - KEAP1 C carrier	NRF2 CC - KEAP1 C carrier	NRF2 T carrier - KEAP1 GG	NRF2 CC - KEAP1 GG	***P* values (F values)**
Schizophrenia	(n = 51)	(n = 76)	(n = 20)	(n = 36)	
Full-scale IQ	81.4 ± 16.5	85.4 ± 19.3	87.1 ± 17.2	91.7 ± 15.9	0.13 (1.9)
Verbal Comprehension	89.7 ± 17.0	92.5 ± 17.7	94.7 ± 17.7	97.4 ± 12.3	0.35 (1.1)
Perceptual Organization	84.1 ± 16.3	86.8 ± 20.2	86.2 ± 16.9	91.2 ± 18.9	0.48 (0.8)
Working Memory	84.0 ± 17.8	87.4 ± 16.9	91.8 ± 14.1	97.7 ± 17.3	**0.0036 (4.7)**
Processing Speed	73.5 ± 16.1	80.6 ± 16.0	71.9 ± 14.0	85.9 ± 13.7	**0.0035 (4.7)**
Controls	(n = 123)	(n = 156)	(n = 44)	(n = 62)	
Full-scale IQ	110.6 ± 11.5	109.2 ± 12.2	112.1 ± 12.6	110.7 ± 13.4	0.17 (1.7)
Verbal Comprehension	108.9 ± 12.5	107.2 ± 13.5	109.3 ± 11.3	108.3 ± 14.5	0.31 (1.2)
Perceptual Organization	107.6 ± 13.1	106.9 ± 12.8	107.9 ± 14.3	107.5 ± 12.3	0.85 (0.3)
Working Memory	106.5 ± 13.7	106.4 ± 15.1	108.6 ± 15.5	108.4 ± 16.6	0.41 (1.0)
Processing Speed	109.3 ± 14.0	108.7 ± 13.7	111.0 ± 14.5	111.5 ± 13.3	0.35 (1.1)

Data are the mean ± SD. Significant *P* values are shown in boldface and underlined.
