# Supplementary material for: Dietary Intake of Sulforaphane-Rich Broccoli Sprout Extracts during Juvenile and Adolescence Can Prevent Phencyclidine-Induced Cognitive Deficits at Adulthood
Source: PLoS One. 2015 Jun 24;10(6):e0127244. doi: 10.1371/journal.pone.0127244 (PMC4479552; doi:10.1371/journal.pone.0127244)
Supplement: S2 Table — (PDF) [file pone.0127244.s002.pdf]

Table S2. Genetic interaction between rs10930781 and rs11545829 on intellectual functions in patients with schizophrenia and healthy subjects

Variables	NRF2 T carrier - KEAP1 T carrier	NRF2 CC - KEAP1 T carrier	NRF2 T carrier - KEAP1 CC	NRF2 CC - KEAP1 CC	*P* values (F values)
Schizophrenia	(n = 38)	(n = 54)	(n = 33)	(n = 58)	
Full-scale IQ	81.5 ± 16.6	86.4 ± 19.7	84.7 ± 17.2	88.4 ± 17.4	0.44 (0.9)
Verbal Comprehension	89.8 ± 17.4	93.9 ± 18.1	92.5 ± 17.2	94.2 ± 14.4	0.82 (0.3)
Perceptual Organization	84.0 ± 17.2	88.7 ± 20.8	85.4 ± 15.7	87.7 ± 19.1	0.57 (0.7)
Working Memory	83.2 ± 17.4	86.6 ± 16.4	89.7 ± 16.4	94.5 ± 18.0	**0.012 (3.8)**
Processing Speed	74.9 ± 16.9	80.5 ± 16.5	71.0 ± 13.7	83.9 ± 14.4	**0.0024 (5.0)**
Controls	(n = 92)	(n = 118)	(n = 75)	(n = 100)	
Full-scale IQ	109.4 ± 11.4	109.6 ± 12.7	112.9 ± 12.0	109.8 ± 12.5	0.07 (2.4)
Verbal Comprehension	108.4 ± 12.9	107.7 ± 14.0	109.8 ± 11.2	107.3 ± 13.5	0.35 (1.1)
Perceptual Organization	106.7 ± 13.0	107.5 ± 12.8	108.9 ± 13.9	106.6 ± 12.5	0.55 (0.7)
Working Memory	104.7 ± 12.8	107.2 ± 15.3	109.9 ± 15.2	106.8 ± 15.9	0.10 (2.1)
Processing Speed	107.9 ± 13.8	108.9 ± 14.1	111.9 ± 14.2	110.2 ± 13.1	0.20 (1.6)

Data are the mean ± SD. Significant *P* values are shown in boldface and underlined. *Post hoc* analysis on working memory found that patients with CC–CC of rs10930781 and rs11545829 scored higher than those with T carrier–T carrier (*P* = 0.0018) and those with CC–T carrier (*P* = 0.021). On the other hand, *post hoc* analysis on processing speed found that patients with CC–CC scored higher than those with T carrier–T carrier (*P* = 0.023) and those with T carrier–CC (*P* = 0.00031), and patients with CC–T carrier scored higher than those with T carrier–CC (*P* = 0.0089).
