# Supplementary material for: Dietary Intake of Sulforaphane-Rich Broccoli Sprout Extracts during Juvenile and Adolescence Can Prevent Phencyclidine-Induced Cognitive Deficits at Adulthood
Source: PLoS One. 2015 Jun 24;10(6):e0127244. doi: 10.1371/journal.pone.0127244 (PMC4479552; doi:10.1371/journal.pone.0127244)
Supplement: S3 Table — (PDF) [file pone.0127244.s003.pdf]

Table S3. Effect of rs10930781 genotype on intellectual ability

Variables	T carrier	CC	*P* values (F values)
Schizophrenia	(n = 71)	(n = 112)	
Full-scale IQ	83.0 ± 16.8	87.4 ± 18.5	0.15 (2.1)
Verbal Comprehension	91.1 ± 17.2	94.0 ± 16.2	0.42 (0.6)
Perceptual Organization	84.7 ± 16.4	88.2 ± 19.8	0.20 (1.7)
Working Memory	86.2 ± 17.1	90.7 ± 17.6	0.081 (3.1)
Processing Speed	73.1 ± 15.5	82.3 ± 15.4	**0.00074 (11.8)**
Controls	(n = 167)	(n = 218)	
Full-scale IQ	111.0 ± 11.8	109.6 ± 12.6	0.12 (2.4)
Verbal Comprehension	109.0 ± 12.1	107.5 ± 13.7	0.11 (2.6)
Perceptual Organization	107.7 ± 13.4	107.1 ± 12.6	0.50 (0.5)
Working Memory	107.0 ± 14.1	107.0 ± 15.5	0.78 (0.1)
Processing Speed	109.7 ± 14.1	109.5 ± 13.6	0.70 (0.1)

Data are the mean ± SD. Significant *P* values are shown in boldface and underlined.
