# Supplementary material for: Dietary Intake of Sulforaphane-Rich Broccoli Sprout Extracts during Juvenile and Adolescence Can Prevent Phencyclidine-Induced Cognitive Deficits at Adulthood
Source: PLoS One. 2015 Jun 24;10(6):e0127244. doi: 10.1371/journal.pone.0127244 (PMC4479552; doi:10.1371/journal.pone.0127244)
Supplement: S4 Table — (PDF) [file pone.0127244.s004.pdf]

Table S4. Effect of rs1048290 genotype on intellectual ability

**Variables**	**C carrier**	**GG**	***P* values (F values)**
Schizophrenia	(n = 127)	(n = 56)	
Full-scale IQ	83.8 ± 18.3	90.0 ± 16.4	0.055 (3.7)
Verbal Comprehension	91.3 ± 17.4	96.4 ± 14.4	0.10 (2.7)
Perceptual Organization	85.7 ± 18.7	89.4 ± 18.2	0.34 (0.9)
Working Memory	86.0 ± 17.3	95.6 ± 16.3	**0.0010 (11.1)**
Processing Speed	77.7 ± 16.4	80.9 ± 15.2	0.34 (0.9)
Controls	(n=279)	(n=106)	
Full-scale IQ	109.8 ± 11.9	111.2 ± 13.0	0.11 (2.5)
Verbal Comprehension	108.0 ± 13.0	108.7 ± 13.2	0.33 (1.0)
Perceptual Organization	107.2 ± 12.9	107.7 ± 13.1	0.57 (0.3)
Working Memory	106.5 ± 14.5	108.5 ± 16.1	0.10 (2.6)
Processing Speed	109.0 ± 13.8	111.3 ± 13.7	0.086 (3.0)

Data are the mean ± SD. Significant *P* values are shown in boldface and underlined.
