# Supplementary material for: Dietary Intake of Sulforaphane-Rich Broccoli Sprout Extracts during Juvenile and Adolescence Can Prevent Phencyclidine-Induced Cognitive Deficits at Adulthood
Source: PLoS One. 2015 Jun 24;10(6):e0127244. doi: 10.1371/journal.pone.0127244 (PMC4479552; doi:10.1371/journal.pone.0127244)
Supplement: S5 Table — (PDF) [file pone.0127244.s005.pdf]

Table S5. Effect of rs11545829 genotype on intellectual ability

**Variables**	**T carrier**	**CC**	***P* values (F values)**
Schizophrenia	(n = 92)	(n = 91)	
Full-scale IQ	84.3 ± 18.5	87.1 ± 17.3	0.41 (0.7)
Verbal Comprehension	92.2 ± 17.8	93.6 ± 15.4	0.75 (0.1)
Perceptual Organization	86.8 ± 19.4	86.9 ± 17.9	0.86 (<0.1)
Working Memory	85.2 ± 16.8	92.7 ± 17.5	**0.0040 (8.5)**
Processing Speed	78.2 ± 16.8	79.2 ± 15.4	0.83 (<0.1)
Controls	(n = 210)	(n = 175)	
Full-scale IQ	109.5 ± 12.1	111.1 ± 12.4	0.089 (2.9)
Verbal Comprehension	108.0 ± 13.5	108.3 ± 12.6	0.60 (0.3)
Perceptual Organization	107.1 ± 12.9	107.6 ± 13.1	0.63 (0.2)
Working Memory	106.1 ± 14.3	108.2 ± 15.7	0.10 (2.7)
Processing Speed	108.5 ± 14.0	110.9 ± 13.5	0.059 (3.6)

Data are the mean ± SD. Significant *P* values are shown in boldface and underlined.
